# The Safety and Performance of a Novel Extracorporeal Membrane Oxygenation Device in a Long-Term Ovine Model

**DOI:** 10.3390/arm93050034

**Published:** 2025-09-09

**Authors:** Yongchao Li, Lei Cai, Jia Huang, Hongbin Gao, Zhongqiang Huang, Yalun Guan, Yunfeng Li, Shuhua Liu, Shi Liang, Summer Xiatian Li, Hongzhou Lu, Ge Li, Yijiang Li, Yu Zhang

**Affiliations:** 1 Guangdong Provincial Biotechnology Research Institute (Guangdong Provincial Laboratory Animals Monitoring Center), No.11, Fengxin Road, Huangpu District, Guangzhou 510663, China; ycl@gdlami.com (Y.L.); cail@gdlami.com (L.C.); ghb@gdlami.com (H.G.); hzq@gdlami.com (Z.H.); gyl@gdlami.com (Y.G.); lyf@gdlami.com (Y.L.); shuhualiu@gdlami.com (S.L.); liangshi@gdlami.com (S.L.); 2 Guangzhou National Laboratory, No. 9, Xingdao Ring North Road, Guangzhou International Bio Island, Huangpu District, Guangzhou 510005, China; 3 National Clinical Research Center for Infectious Diseases, The Third People’s Hospital of Shenzhen, 29 Bulan Road, Longgang District, Shenzhen 518112, China; hjsunnyvale@hotmail.com; 4 Guangdong Organ Support Engineering Technology Research Center, Bldg. 6, Baoxing Wisdom City, No. 650 Zhoushi Road, Bao’An District, Shenzhen 518126, China; summerxiatian.li@icloud.com

**Keywords:** ECMO support, ovine model, preclinical long-term evaluation, critical care, proteomics

## Abstract

**Highlights:**

**What are the main findings?**

- In this work, we evaluated a novel extracorporeal membrane oxygenation (ECMO) device (Lifemotion^®^, Chinabridge, China) with veno-nenous (VV) and veno-arterial (VA) ECMO in a ovine model for assessing the safety and performance of the ECMO device and found the ECMO device could support in keeping experimental sheep alive for 14 days and showed a comparable performance as the marketed product (Novalung^®^ Xlung™ kit 230, Xonis, Germany).

**What is the implication of the main finding?**

- This research also supported the preclinical animal trials of Lifemotion^®^, the first ECMO device approved in China for market launch.

**Abstract:**

Since extracorporeal membrane oxygenation (ECMO) is primarily used for patients in a high-risk state and is an invasive procedure, its unique application scenarios make it difficult to recruit suitable cases for clinical trials. Therefore, large animal models have become one of the most important models for preclinical evaluation of the safety and effectiveness of ECMO. This study aims to assess the safety and performance of a novel portable ECMO device with Small-tail Han sheep. Fifteen sheep were divided into a test group (LIFEMOTION, Chinabridge, Shenzhen, China) and control group (NOVALUNG XLUNG kit 230, Xonis, Heilbronn, Germany) with veno-venous ECMO (VV-ECMO) and veno-arterial ECMO (VA-ECMO) modes. Tracheal intubation, arteriovenous access, and ECMO support were performed. Vital signs and blood laboratory tests of the subjects were monitored and recorded. The main organs were examined pathologically at the end of day fourteen. The serum protein expression profile was analyzed by protein quantification techniques. All sheep were successfully weaned from ECMO without transfusion or cannula complications. No significant differences were observed between the two groups in terms of vital signs, oxygenation, hemodynamic stability, and physiological function (*p* > 0.05). According to the serum protein expression profile, no significant biomarkers associated with ECMO clinical complications were identified. The LIFEMOTION ECMO device demonstrated good safety and efficacy.

## 1. Introduction

Extracorporeal membrane oxygenation (ECMO) serves as an important equipment for the treatment of critically ill patients and provides a bridge for the recovery or transplantation of natural organs [1,2]. It has played a significant role in the emergency treatment of critically ill patients during the COVID-19 pandemic [3,4]. With advances in ECMO technology and the COVID-19 pandemic globally, ECMO support was widely applied to acute respiratory distress syndrome (ARDS), heart failure, respiratory failure, and organ transplantation and improved survival [5,6,7].

According to different ECMO configurations and cannulation strategies, ECMO support includes veno-venous ECMO (VV-ECMO) and veno-arterial ECMO (VA-ECMO) [8]; in VV-ECMO, venous blood is withdrawn from the venous system through a drainage cannula, and oxygenated blood is reinfused into the venous system crossing the tricuspid valve into the right ventricle, providing primarily respiratory support; during VA-ECMO, the pump returns oxygenated blood to the arterial system, providing circulatory support [9,10,11].

Since ECMO is primarily used for patients in a high-risk state and is an invasive procedure [12], its unique application scenarios make it difficult to recruit suitable cases for clinical trials. Therefore, large animal models have become one of the most important models for preclinical evaluation of the safety and effectiveness of ECMO [13,14,15], and they are almost irreplaceable. Large animal models play a crucial role in the clinical translation of novel ECMO devices and related application technologies [16,17]. As the National Medical Products Administration (NMPA) guidelines for clinical evaluation and animal studies for ECMO systems highlight, due to the high-risk nature, complex technology, and numerous potential complications of ECMO, laboratory bench testing alone is insufficient to fully evaluate a device’s safety and efficacy. Therefore, preclinical animal studies are essential for providing critical evidence to support product design finalization and to demonstrate the device’s safety and effectiveness before human clinical trials. Currently, the main large animals used in ECMO research with blood volume comparable to humans are sheep [1], cattle [18], pig [19], etc. Due to their large size, cattle are not ideal, while pigs have a smaller size and higher difficulty in arterial cannulation. Sheep are considered a more suitable large animal model. Based on previous studies, research on ECMO in large animals is often short-term (within 7 days) and mostly in single ECMO mode (VV (veno-venous)-ECMO or VA (veno-arterial)-ECMO), with fewer studies on the long-term effects of ECMO on animals [20,21]. In this study, we used Small-tail Han sheep as a large animal model to investigate the long-term performance of a domestically produced novel ECMO device (LIFEMOTION ECMO) under VV and VA-ECMO by coagulation, hematology, blood gas parameters, blood chemistry, and histological and proteomic analysis. We aim to provide a reference for the subsequent clinical translation of ECMO devices and exploration of pathological physiology in large animal models.

## 2. Materials and Methods

### 2.1. Ethics and Animal Groups

Our study is reported in accordance with the ARRIVE guidelines and complies with the Declaration of Helsinki. The investigation conforms to the Guide for the Care and Use of Laboratory Animals published by the US National Institutes of Health (NIH Publication No. 85-23, revised 1985). The study and protocols were authorized by the Institutional Animal Care and Use Committee (IACUC) of the Guangdong Provincial Biotechnology Research Institute (Guangdong Provincial Laboratory Animals Monitoring Center) (IACUC2021164). All sheep were healthy and quantified by the Guangdong Provincial Biotechnology Research Institute (Guangdong Provincial Laboratory Animals Monitoring Center). The 15 male Small-tail Han sheep (China) in this study were about 2–3 years old, and their weight was between 80 to 115 kg, and the mean weight was 102.27 ± 7.91 kg. These sheep were divided into VV-ECMO and VA-ECMO modes by simple random sampling, and each mode included the control group (NOVALUNG XLUNG kit, Xonis, Germany) and the LIFEMOTION group (Chinabridge (Shenzhen) Medical Technology Co., Ltd., Shenzhen, China), respectively (Table 1).

### 2.2. ECMO Device Information

**Figure 1:**
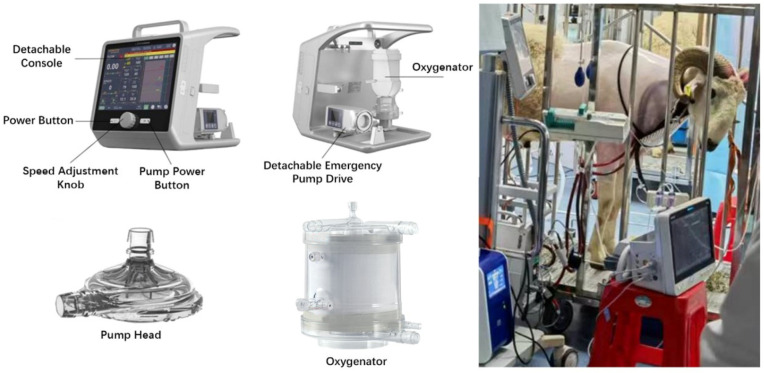
Image of the LIFEMOTION ECMO device components and a sheep subject supported by the LIFEMOTION ECMO. (**Top left panel**): LIFEMOTION ECMO console; (**bottom left panel**): disposable LIFEMOTION pump head and oxygenator. (**Right panel**): sheep subject on the LIFEMOTION ECMO.

The ECMO devices were NOVALUNG XLUNG kit 230 (Xonis, Heilbronn, Germany) and LIFEMOTION (Chinabridge, Shenzhen, China) (Table 1). The LIFEMOTION ECMO device includes a console (LM-ECMO-1000), a disposable pump head (LM-PH-1000), a membrane oxygenator (LM-BGEA-1000), and a disposable membrane oxygenator set (LM-TPS-1000) (Figure 1). The new ECMO device, compared to NOVALUNG XLUNG kit 230, maintains core performance parameters that are at least on par with similar products in the market, while offering the advantages of a lighter weight (11 kg vs. 12 kg), longer battery life (240 min or more vs. 240 min), a smaller priming volume (600 mL vs. 670 mL) and extended usage duration (>7 days vs. 7 days).

### 2.3. Trial Process and Post-Surgical Care

#### 2.3.1. Preparation Before Operation

Before the operation, sheep were fed in cages and adapted for a week. Before surgery, sheep were fasted for 48 h and deprived of water for 24 h.

#### 2.3.2. Priming of the ECMO Devices

Before the operation, it was necessary to perform system testing of the console and disposables. A disposable priming bag was primed with 2 to 3 L of normal saline solution, and the disposable pump head was joined with a tube and an oxygenator, water tank, gas line, and fixed connectors with medical adhesive tape. Then, the water tank was turned onto a warm oxygenator, which was confirmed to have no leakage. The tube and oxygenator were primed with a normal saline solution and by draining air from the pump head, and the tubes were using a static model. The console was turned on, and the rotation speed was adjusted from low to high to remove air bubbles or gas from the oxygenator and tubing. After the air in the circuit was removed, the console was tested, and the rotation speed was increased up to 4000 rotations per minute. The tubing box was opened and checked to observe whether there was leakage in the tubing and the oxygenator during the priming process. After the system was primed, hemostatic clamps were used to clamp the connector of the priming tube for standby.

### 2.4. Operation Procedures

#### 2.4.1. Anesthesia and Endotracheal Intubation

Sheep wool around the neck and both sides of the chest was removed in advance for skin preparation. The sheep were anesthetized with ketamine and xylazine and were put on an operation table after anesthesia. Then, the sheep underwent tracheal intubation using a 13# tracheal tube (Intersurgical, Crane House, UK) by an experienced veterinarian.

After tracheal intubation, the incubation was connected with a breathing machine (SV-600, Mindray, Shenzhen, China) for mechanical ventilation (Vt 10–12 mL/kg, F 12–20 times/min, FiO_2_ 60%, PEEP 5–10 cm H_2_O), maintaining anesthesia by a combined 2–3% isoflurane with intravenous anesthesia (propofol 8–10 mg/kg/h). During the operation, dezocine was administrated intermittently to alleviate pain and maintain the heart rate and mean arterial pressure within the range of 20% of the baseline, the partial pressure of carbon dioxide (pCO2) between 30–45 mmHg, and the potassium levels between 3–4.5 mmol/L, meanwhile adjusting the dosage of anesthetics and the parameters of the breathing machine according to the real-time conditions.

#### 2.4.2. Constructing Arteriovenous Access for Monitoring Vital Signs

An 8.5 f four-chamber central venous catheter (Shenzhen Yixinda Medical New Technology Co., Ltd., Shenzhen, China) was placed in the left jugular vein and a 14 G invasive arterial conduit (Shenzhen Yixinda Medical New Technology Co., Ltd., Shenzhen, China) in the carotid artery, which was connected with electrocardiogram monitoring (ePM-15, Mindray, Shenzhen, China) located at the edge of the ear or the tip of the tongue for continuous monitoring of vital signs.

The procedures of VV-ECMO support were as follows. Sheep were in the supine position. The neck was uncovered completely, the skin was disinfected, and surgical drapes were placed on the neck. The right upper sternal region of the neck was cut into an incision about 6 cm in length; the open skin and hypodermis was cut into, and the jugular vein was dissected and isolated. Then, systemic heparinization was administered with 50,000 units of heparin. After heparinization, lateral wall forceps clamped and held the anterior wall of the jugular vein, and a longitudinal incision of about 8 mm was cut. Then, an artificial blood vessel (Maquet, Rastatt, Germany) with 10 mm in diameter and 5 cm in length was continuously sutured up to the wall of the jugular vein with 4-0 Prolene sutures (Johnson & Johnson, New Brunswick, NJ, USA), making the right jugular vein and the end of the artificial blood vessel join completely. A conducting wire of 30 cm in length was inserted through the artificial blood vessel, which conducted and implanted a 23 Fr ECMO cannula (Medtronic, Minneapolis, MN, USA) with double chambers, a fixed cannula, and the artificial blood vessel with threads. The cannula inserted was dropped into a heparin-saline solution, removed of air completely, and joined with the drainage vessel. The return vessel was primed, carefully evaluated, observed, and confirmed to have no folds in the tubes nor blood oozing in the surgical parts. Finally, the ECMO device was initiated, containing a pump speed with a flow of about 1.5 L.

VA-ECMO support procedures were as follows. Sheep subjects were lying on the right side and uncovered the left skin in the chest. The fourth intercostal space under the left scapula was cut with an incision of 8 cm close to the next costa, opening the skin and hypodermis fascia, muscle, and up to the left thorax from the fourth intercostal upside. After into the left thorax, tidal volume was reduced to avoid pulmonary injury, and then the left upper lobe of the lung was pulled down from the side of the spine and wrapped with a wet gauze pad to protect it, downwardly exposing the left pericardium, extracardiac vessel, and descending aorta. Then, the pericardium was suspended after a 4 cm incision was made along with the axis of the left pulmonary artery of the sympathetic nerve and thoracic nerve. The pulmonary artery, about 2–2.5 cm in diameter, was observed, and an isolated pulmonary artery fixed with a thick, disinfected silk thread followed the descending aorta above the reverse laryngeal nerve and thoracic nerve. The thorax cavity and pleura were cut and opened into the descending aorta, and then the descending aorta was isolated to about 3–4 cm in length. The descending aorta was thickened and fixed through the posterior wall of the descending aorta with pliers, which was conducted by right-angle pliers. Systemic heparinization was performed with 5000 units.

After heparinization, the arterial pulmonary artery was clamped with forceps and an incision about 10 mm in length; an artificial blood vessel of 10 mm in diameter and 6 cm in length with a 4-0 Prolene suture was placed up to the anterior wall of the pulmonary artery and joined with the artificial blood vessel with the pulmonary artery, and after joining, pliers were opened, releasing the pulmonary artery, and no signs of leakage and bleeding were also confirmed. Then an incision about 8 cm in diameter was incised in the descending aorta and was joined with the end of an artificial blood vessel of 8 mm in diameter and 6 cm in length. An incision was cut between the sixth and seventh costa, constructing a subcutaneous channel and embedding 22 Fr and 24 Fr single lumen cannulas (Medtronic, Minneapolis, MN, USA) conducted through guide wires, and then fixed to cannula with artificial blood vessels of descending aorta and pulmonary artery, respectively. After fixation, the heparin-saline solution was injected into the cannula, any air bubbles were removed, and the cannula was connected to primed venous and arterial tubes; a clear cannula was maintained with no oozing in the surgical region. After turning on the ECMO, the speed and flow was adjusted to about 0.8–1.5 L. Closed thoracic drainage was performed routinely, and the chest wall was sutured, fixing cannulas to the chest wall.

### 2.5. Standard Management After Operation

#### 2.5.1. Critical Management

After the operation, the endotracheal tube was connected with a breathing balloon and put into a specialized cage for monitoring vital signs and blood gas. Meanwhile, the respiratory state and interior circumstances of sheep subjects were monitored and dealt with promptly. Before, sheep could stand by themselves, be cared for, and be managed by a special nurse. Under anesthesia, the position of sheep could cause pulmonary atelectasis with a large possibility. Therefore, assistant standing was necessary. After awakening from anesthesia, given the long-term survival, awake ECMO was performed on the sheep, extubating the tracheal tube as soon as possible and giving mask oxygen when recovering spontaneous respiration. After extubating the tube, consciousness, breath, and movements were carefully watched. Once the sheep could stand, general care was provided.

#### 2.5.2. General Care and Treatment

After waking up, sheep could drink water first and then feed on food, including hay and pellet feedstuff. After the operation, symptoms of thrombosis, hemolysis, limb ischemia, bleeding, infection, and equipment failure were monitored, recorded, and dealt with promptly. During the awake process, sheep were apt to pull the cannulas out from vessels, chew the cannulas apart, etc. Therefore, a large Elizabeth circle fixed sheep in the neck when they recovered partial activity. Except for necessary cannula fixation of ECMO equipment, other tubes or cannulas were kept loosely from knotting or fall-down. Moreover, the arteriovenous cannula line and monitoring lines were specially protected with a plastic cover. After the operation, sheep were administrated for sedation and anti-infection with antibiotics, and their physical condition was monitored daily. The daily observation included a healthy state, activity, dairy diet, etc. A series of physiological parameters were measured at scheduled time points to maintain stable vital signs in the sheep.

To minimize secondary infections, we maintained dry housing conditions and promptly removed fecal matter. Additionally, targeted antibiotics and sulfonamides were administered based on hematology results to control infection.

#### 2.5.3. The Management of Bleeding and Anticoagulation

The venous vessel was prone to tearing and bleeding during the cannula insertion process. ECMO circulates cannulas located in the neck, and the circulated cannulas may deform because of daily pulls and observation. The insertion incision would cause hemorrhage, even with cannula removal. A minor amount of bleeding at the incision site was treated by squeezing and suturing while adjusting the heparin dose. When a large amount of bleeding occurs, the incision should be re-sutured to stop bleeding and re-tied at the bleeding site; even re-inserting the cannula should be considered. During ECMO running, the balance of the coagulation state was necessary, maintaining a higher anticoagulation state for preventing thrombosis and a rational coagulation state for no bleeding in incisions or tissues, which means increasing the measuring frequency of ACT and other coagulation indexes. According to the coagulation state, adjusting heparin dosage prevents thrombin in the cannula, maintaining the anticoagulation balance in equipment during the whole trial period. If the ECMO-fixed cannula was wrong, sheep would be anesthetized and have re-fixed cannulas; otherwise circulating channels would fall out from the sutured incision, resulting in a mismatch of inserted cannulas or shafted cannula outlets and blocked vessels.

#### 2.5.4. Post-Operation Care, Monitoring, and Data Collection

Monitoring the vital signs and ECMO parameters (including speed, flow rate, pre-pump pressure, post-pump pressure, and post-oxygenator pressure) was in real-time, and the collected blood was evaluated and analyzed for blood gas, complete cell count, and blood biochemistry, and a coagulation test was performed every day.

#### 2.5.5. Histological Analysis

Sheep subjects were weaned off ECMO at the termination time and were euthanized, with potassium chloride (100 mg/kg) under the sedation of propofol (20 mg/kg), and dissected based on gross anatomy conducted by pathologists. Main organ (lung, heart, liver, kidney, spleen, cerebellum, brainstem, small intestine, mesenteric lymph nodes, inguinal lymph nodes, and cervical lymph nodes) tissues were examined if there were thrombus or hemorrhage and then photographed. Tissue samples were fixed in 4% paraformaldehyde (Sigma, Ronkonkoma, NY, USA) for pathological detection. Briefly, the tissue blocks embedded in wax were cut and stained with hematoxylin and eosin (Leica SV5030, Wetzlar, Germany). The stained sections were scanned using a Digital Pathology Scanner (Versa 8, Leica, Wetzlar, Germany) and evaluated by professional pathologists.

#### 2.5.6. Data Record and Collection

The hemodynamics, pump flow, and rotation speed were recorded continuously during the trial. Blood samples were collected, and the indicators or parameters of blood gas, hematology, and blood biochemistry were determined, as well as coagulation values and the oxygenator. The values of blood cell count, blood biochemistry, and coagulation were tested at 0 h (pre-operation), within 6 h, 12 h, and 24 h of post-operation, and then twice each day from 2 to 14 days. And the CRP value was measured once each day.

### 2.6. Proteomic Analysis

All 15 animals reached the endpoint of the experiment. Peripheral venous blood samples were collected from all animals at three timepoints: preoperative (0 d), 7 days post-surgery, and 14 days post-surgery. After centrifugation, the serum was obtained and stored at −80 °C for future use. Due to quality issues, samples from only 13 animals were used for subsequent protein sequencing analysis, as shown in Table 2.

Non-targeted protein quantification techniques were used to conduct related analyses. The samples underwent total protein extraction, protein concentration determination, trypsin digestion, liquid chromatography–mass spectrometry, and bioinformatics analysis to identify differentially expressed proteins. The differentially expressed proteins were further subjected to GO functional clustering analysis, KEGG enrichment analysis, protein domain enrichment analysis, and protein–protein interaction network analysis. The specific sequencing work was commissioned to Hangzhou Jingjie Biotechnology Co., Ltd. (Hangzhou, China); detailed procedures can be referred to in the relevant literature. The grouping strategy for bioinformatics analysis is presented in Table 3.

### 2.7. Data Statistics

Due to incomplete data on the physiological and coagulation indexes of sheep in previous reports, we describe the pre-operation data of 15 sheep subjects as the normal range of sheep. The data result showed each 24 h, and the data values showed mean ± standard deviation (SD) and were analyzed by GraphPad Prism 8.0 software with a two-tailed, unpaired *Student’s t-test* and ANOVA analysis. *p* < 0.05 was defined as significant statistically.

## 3. Results

### 3.1. All Sheep Subjects Survived Until the End of the Trial Without Significant Thrombosis

**Figure 2:**
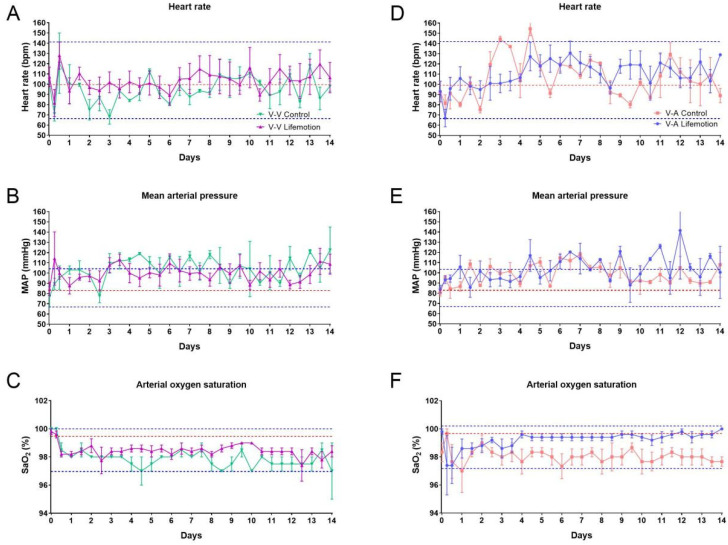
Vital sign values were stable during ECMO, including heart rate, mean arterial pressure, and arterial oxygen saturation. The three dotted lines in the plots represent the pre-surgical highest, average (red), and lowest values. (**A**,**D**) represent the heart rate of the experimental and control groups, respectively. (**B**,**E**) represent the mean arterial pressure of the experimental and control groups, respectively. (**C**,**F**) represent the arterial saturation of the experimental and control groups, respectively. The three dotted lines in the plots represent the pre-surgical highest, average, and lowest values, respectively.

In this study, 15 sheep subjects survived until 14 days (336 h) of the end schedule (Figure 2, right panel). During the trial, all sheep were in good condition after recovery from anesthesia, without transfusion. None of the sheep had symptoms of unconsciousness and dyspnea, and no signs of oxygenator membrane rupture resulting in gas–blood mixing were observed. Meanwhile, there were no serious adverse events due to the leakage at the oxygenator outlet and the heater-cooler, and no disposable items were changed due to thrombosis. Through observation of the equipment pipelines and tubing, no apparent thrombus formation was observed. Moreover, all sheep were successfully weaned off ECMO support without hemolysis, thrombosis, or hemorrhage.

### 3.2. The Sheep Subjects of the LIFEMOTION Group Showed Stable Vital Signs During ECMO Support

Vital signs are regarded as an essential part of monitoring patients in intensive care units, and significant changes in the vital signs imply probable clinical deterioration. Thus monitoring vital signs is critical for timely intervention [22]. In this study, the vital signs including heart rate (HR), mean arterial pressure (MAP), and arterial oxygen saturation (SO2) were determined with a monitor continuously. The HR, MAP, and SO2 values of the 15 sheep at pre-operation were 98.67 ± 18.90 beats per minute, 82.47 ± 10.36 mmHg, and 99.53 ± 0.83% (Supplementary Table S1).

During VV-ECMO and VA-ECMO, the LIFEMOTION and control groups showed comparable HR and MAP values. Meanwhile, the LIFEMOTION group showed a significantly higher SO2 than the control group during VA-ECMO from 4 to 14 days (Figure 2, Supplementary Tables S2–S5).

Compared to pre-operation values, the HR values of the sheep in VV-ECMO and VA-ECMO waved around the average value of pre-operation, and most HR values were in the range of pre-operation values. MAP values of the subjects during ECMO were higher than the pre-operation average, and MAP values at several time points were out of the pre-operation range. Although SO_2_ values were lower than the average pre-operation values, the SO_2_ values of the sheep were above 97% (Figure 2). A total of 95 to 100 percent of SO_2_ values are considered normal clinically [23]. Thus, HR and SaO_2_ values during ECMO were in the normal range, while several MAP values above the normal range were due to ECMO support or care management.

**Figure 3:**
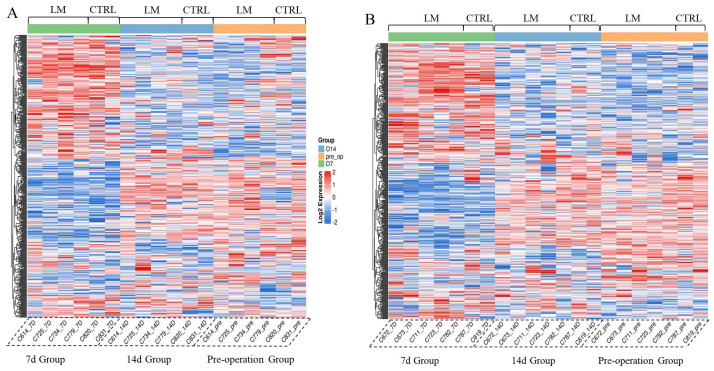
Heat map visualizing the differential expression of proteins across Pre-operation, 7 d post-surgery, and 14 d post-surgery groups in ECMO. Note: (**A**) represents VA-ECMO; (**B**) represents VV-ECMO. LM represents LIFEMOTION group; CTRL represents control group. Red represents high expression proteins, blue represents low expression proteins, and gray represents proteins that cannot be quantified in the corresponding sample. The results showed that the protein expression profiles of the LIFEMOTION group were similar to those of the control group at each time point.

**Figure 4:**
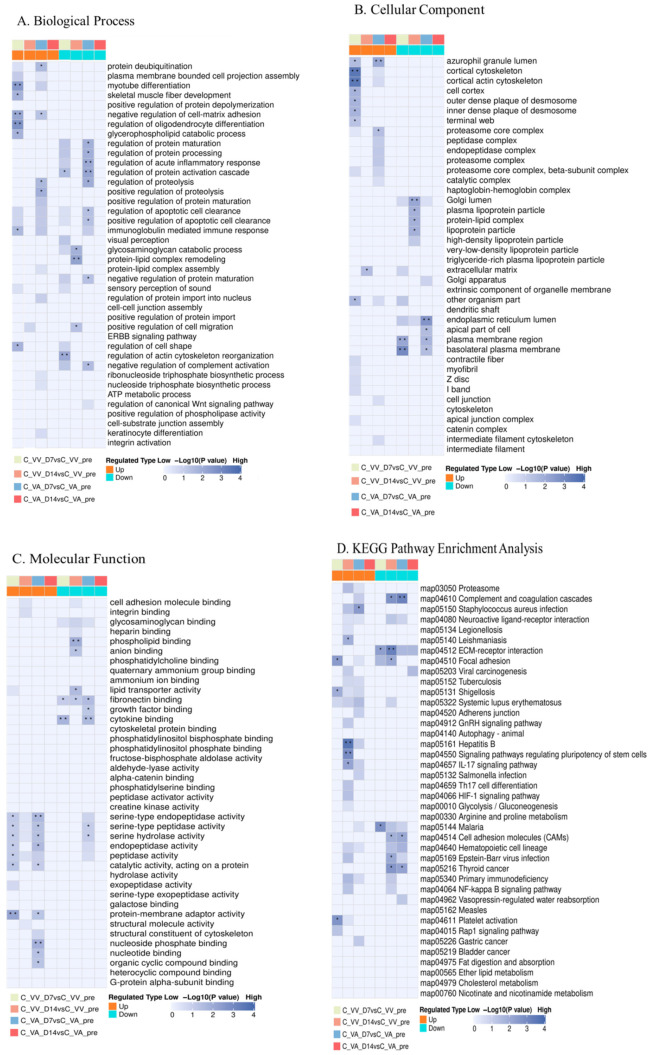
Differential expression protein GO and KEGG enrichment analysis. Note: (**A**) represents biological process classification in GO analysis, (**B**) represents cellular component classification in GO analysis, (**C**) represents molecular function classification in GO analysis, and (**D**) represents KEGG analysis; * indicates a significant difference (*p* < 0.05), ** indicates an extremely significant difference (*p* < 0.01).

Proteomics technology was used to analyze the sheep serum samples. The results showed that the protein expression profiles of the LIFEMOTION group were similar to those of the control group at each time point (Figure 3). At 7 days after surgery, both the LIFEMOTION group and the control group animals exhibited significant differences in serum protein expression profiles compared to the pre-operation group. However, 14 days after surgery, regardless of the VA or VV-ECMO, the serum protein expression profiles of the LIFEMOTION group and the control group animals recovered to pre-operation levels (Figure 3). GO and KEGG analysis were performed on differentially expressed proteins. No significant ECMO-related pathways were found in the GO and KEGG classifications (Figure 4).

The results of the proteomics experiment further confirmed that the LIFEMOTION group device had minimal impact on the protein expression profiles of large animal blood and demonstrated overall good safety.

### 3.3. Sheep Subjects of the LIFEMOTION Group Had Satisfying Oxygenation During ECMO Support

Arterial blood gas measurements are a mainstay of clinical care and are used to assess the abnormalities in pulmonary gas exchange [24]. The results of blood gas values are used to diagnose acid–base balance, oxygenation, and ventilation disorders, provide important information on the state of the patient’s oxygenation and gas exchange, and regulate the pump and the oxygenator parameters during ECMO support [24,25]. In this study, blood gas parameters were determined, including partial pressure of oxygen (PO2), partial pressure of carbon dioxide (PCO2), and lactic acid (LAC). The pre-operation PO2, PCO2, and LAC of sheep subjects were 231.60 ± 145.04 mmHg, 42.68 ± 14.35 mmHg, 0.60 ± 0.34 mmol/L, respectively.

**Figure 5:**
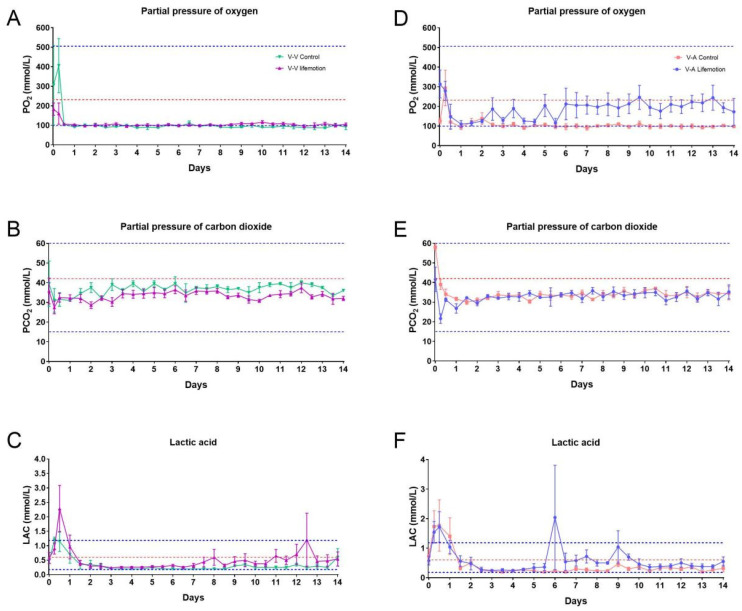
Blood gas values showed the LIFEMOTION group had a better gas exchange during VA-ECMO support. The three dotted lines in the plots represent the pre-surgical highest, average (red), and lowest values. (**A**,**D**) represent the partial pressure of oxygen for the experimental and control groups, respectively. (**B**,**E**) represent the partial pressure of carbon dioxide for the experimental and control groups, respectively. (**C**,**F**) represent the lactic acid levels for the experimental and control groups, respectively. The three dotted lines in the plots represent the pre-surgical highest, average, and lowest values, respectively.

During VV-ECMO, the LIFEMOTION group showed slightly higher PO2 than the control group and significantly higher PO2 than the control group during VA-ECMO. Meanwhile, the LIFEMOTION group showed slightly lower PCO2 during VV-ECMO and comparable PCO2 during VA-ECMO (Figure 5, Supplementary Tables S2–S5). The LAC values of the LIFEMOTION group were higher than those of the control group, but the LAC values of the control group and the LIFEMOTION group were mostly kept within the normal range during ECMO (Figure 5). These results suggested the sheep had adequate oxygenation.

For the physiological role of hematology in assessing general health and disorders, blood cell count and blood biochemistry were tested in the sheep subjects for evaluating general health. The animals of control and LIFEMOTION groups showed comparable values in blood cell count and blood biochemistry (Supplementary Tables S2–S5). We also noted several higher variable indexes (Figure 5C,F), which may be due to the stress response after the surgery and post-surgery management.

### 3.4. Sheep Subjects Showed Adequate Anticoagulation Management During ECMO Support

Clinically, patients supported by ECMO are at an increased risk of developing significant coagulopathy, and blood exposure to the ECMO circuit’s artificial surface can activate the coagulation–fibrinolysis system and inflammatory response [26]. Therefore, anticoagulation monitoring is essential for ECMO patients or sheep during ECMO [1,27,28].

In the previous document, the FIB value is about 1.97 ± 0.55 g/L, and the APTT is 33.00 ± 6.79 s [29]. In this research, the pre-operation coagulation indicators include activated clotting time (ACT 221.69 ± 69.82 S), prothrombin time (PT 17.07 ± 5.45 S), activated partial thromboplastin time (APTT 63.24 ± 59.88 S), and fibrinogen (FIB 0.85 ± 0.28 g/L); and the sheep subjects had a lower FIB and higher APTT than those of previous reported [29].

**Figure 6:**
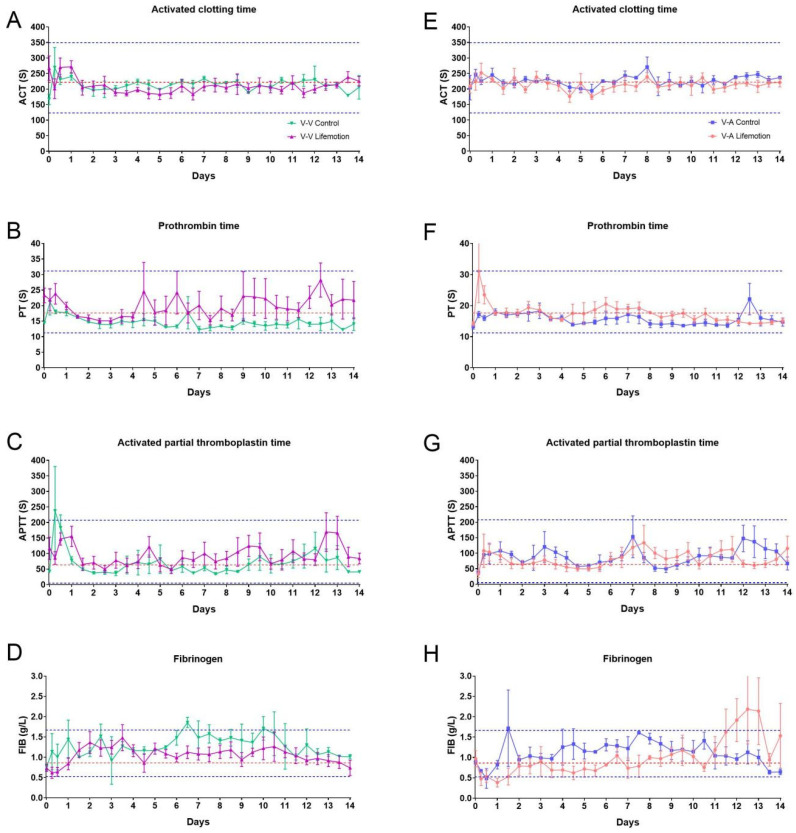
Sheep subjects showed satisfactory management of anti-coagulation during ECMO. The three dotted lines in the plots represent the pre-surgical highest, average (red), and lowest values. (**A**,**E**) represent the activated clotting time for the experimental and control groups, respectively. (**B**,**F**) represent the prothrombin time for the experimental and control groups, respectively. (**C**,**G**) represent the activated partial thromboplastin time for the experimental and control groups, respectively. (**D**,**H**) represent the fibrinogen levels for the experimental and control groups, respectively. The three dotted lines in the plots represent the pre-surgical highest, average, and lowest values, respectively.

The LIFEMOTION group showed comparable ACT and FIB and slightly higher PT and APTT than the control group in VV-ECMO support. Meanwhile, the animals of the LIFEMOTION group showed dramatic changes in APTT and FIB during VA-ECMO. Although the coagulation indicators were variable during ECMO, they were in the pre-operation range (Figure 6). ACT values were within the baseline range of about 220 s organs [1,29]. Therefore, these results suggested that the sheep subjects had satisfactory anticoagulation management during ECMO support.

### 3.5. The Oxygenator of the LIFEMOTION Group Showed Comparable Performance in Oxygenation to the Control Group During ECMO

**Figure 7:**
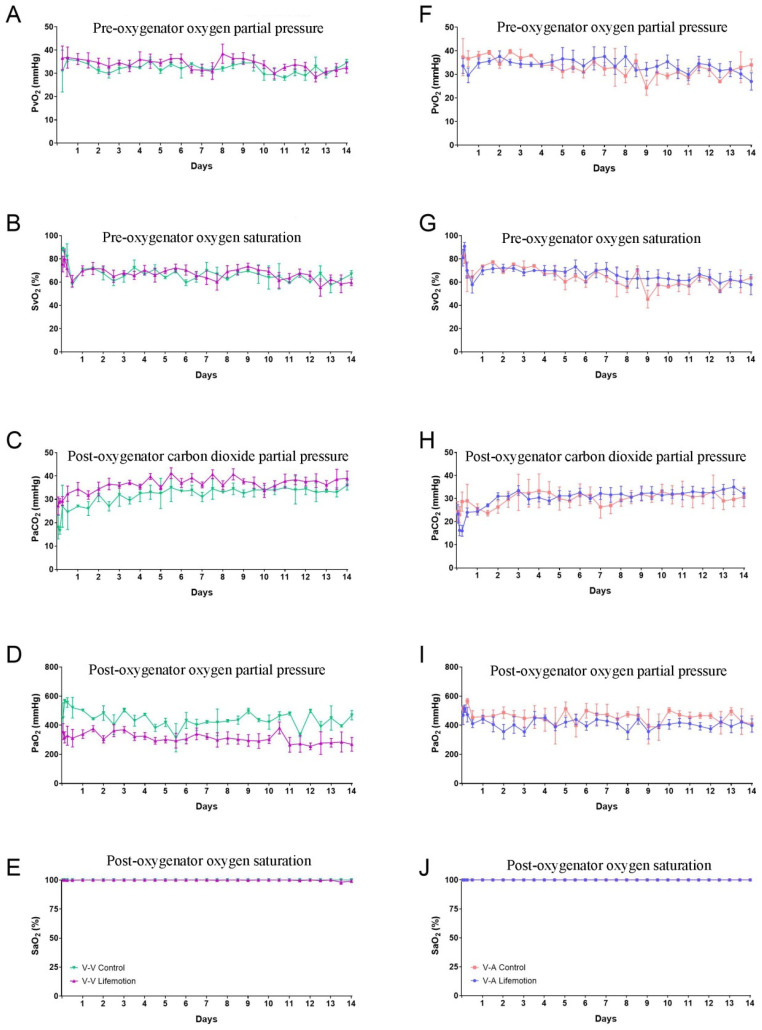
The oxygenators of the LIFEMOTION group had a comparable oxygenating efficacy compared with that of the control group. Abbreviations: PvO_2_, pre-oxygenator partial pressure of oxygen; SvO_2_, pre-oxygenator oxygen saturation; PaCO_2_, post-oxygenator partial pressure of carbon dioxide; PaO_2_, post-oxygenator partial pressure of oxygen; SaO_2_, post-oxygenator oxygen saturation. (**A**,**F**) represent the pre-oxygenator oxygen partial pressure for the experimental and control groups, respectively. (**B**,**G**) represent the pre-oxygenator oxygen saturation for the experimental and control groups, respectively. (**C**,**H**) represent the post-oxygenator carbon dioxide partial pressure for the experimental and control groups, respectively. (**D**,**I**) represent the post-oxygenator oxygen partial pressure for the experimental and control groups, respectively. (**E**,**J**) represent the post-oxygenator oxygen saturation for the experimental and control groups, respectively. The three dotted lines in the plots represent the pre-surgical highest, average, and lowest values, respectively.

Oxygenator performance was evaluated through partial pressure of arterial oxygen after the oxygenator (PaO_2_), partial pressure of carbon dioxide after the oxygenator (PaCO_2_), and oxygen saturation after the oxygenator (SaO_2_) [20,27,30]. During ECMO support, the pressure of oxygen before the oxygenator (PvO_2_) and oxygen saturation before the oxygenator (SvO_2_) were comparable between control and LIFEMOTION groups (Figure 7A,F,B,G and Supplementary Tables S1–S4). In VV-ECMO and VA-ECMO support, PaO_2_ was above 200 mmHg, and PaCO_2_ was below 45 mmHg (VV-ECMO 29.2-41.4 mmHg, VA-ECMO 24.4–34.0 mmHg) during ECMO (Figure 7C,H,D,I). The SaO_2_ of the sheep was about 100% through oxygenation of the oxygenator. There was no significant difference between the control and LIFEMOTION groups (Figure 6E,J). The results suggested the sheep of the LIFEMOTION group had good oxygenation, and the LIFEMOTION ECMO device showed a parallel oxygenation capacity with the control ECMO.

### 3.6. Pathological Analysis Revealed No Major Complications or Organ Damage

**Figure 8:**
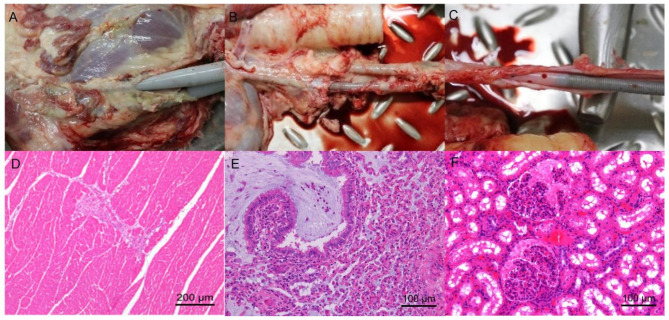
The pathological results. (**A**) Cannulation site without shifting or falling of the cannula tubes and no obvious signs of thrombosis, hematomas, or bleeding. (**B**) Correct cannulation sites; jugular vein cannula positioned correctly; superior vena cava was visible without obvious cannula shifting or displacement of cannula tubes. (**C**) Cannula tube in the blood vessel without obvious thrombosis or hematoma. (**D**) One sheep with a necrosis of cardiac muscle tissue (S12, LIFEMOTION group of VA-ECMO support). (**E**) The mucus accumulation with neutrophils in moderate bronchus of lung (S2 control group of VV-ECMO support). (**F**) The lumen expansion of glomerular capsule in kidney (S10, control group of VA-ECMO support).

All 15 sheep survived for 14 days until the trial’s end. After being weaned, the sheep were euthanized and necropsied by pathologists. The cannula sites were visualized, and the placements were confirmed correct by pathologists, without cannula shifting and falling. The tissues at the cannulation site showed no signs of thrombosis, hematoma, or bleeding (Figure 8A–C).

The necropsy was performed by pathologists, and the main organs examined, such as the brain, heart, liver, spleen, lung, and kidney, were not found to have obvious hematomas and bleeding sites. In addition to the background pathological changes, pathological results showed one sheep with mucus accumulation with neutrophils in moderate bronchus in the control group and one sheep with necrosis of cardiac muscle in the LIFEMOTION group, and one sheep kidney tissue showed necrosis and infiltration of inflammatory cells in the kidney. However, we could not confirm if there was a relationship between ECMO support and pathological changes (Figure 8D–F).

### 3.7. The Novel Long-Term ECMO Device Had a Negligible Impact on the Animal’s Serum Protein Profile

Using protein profiling test results, differentially expressed proteins among different groups were selected, with a fold change exceeding 3-fold as the significant upregulation or downregulation screening threshold. In the VA-ECMO, compared with the preoperative group, the LIFEMOTION group had fewer upregulated and downregulated expressed proteins at 7 d and 14 d after initiating ECMO, especially at 14 d, with only 12 upregulated proteins and 7 downregulated proteins. According to the major types of complications during ECMO support published by the Extracorporeal Life Support Organization (ELSO) (coagulation, hemolysis, infection, inflammation-related, organ damage, etc.) (Supplementary Table S6), an analysis of potential protein markers in the differentially expressed proteins associated with ECMO complications was performed (Table 4). At 7 d after initiation, one coagulation-related highly expressed protein (F13A), five inflammation-related proteins, and two organ damage-related proteins were identified. At 14 d, compared with the 7 d data, only one potentially muscle-repair-related highly expressed protein CMK (VA-ECMO can cause trauma to local muscles) remained, and the Hemoglobin subunit beta protein, which may be clinically insignificant, was newly identified. This is because the results of routine blood tests showed no statistically significant difference in indicators related to hemolysis-associated free hemoglobin (*p* < 0.05). Protein profiling test results showed that, in the VA mode at 7 d after surgery, some inflammation-related indicators proteins could be identified, and at 14 d, they tended to be normal. No clinically significant biomarker proteins associated with ECMO were observed.

In VV-ECMO, compared to the preoperative group, the LIFEMOTION group had fewer upregulated or downregulated proteins at 7 d and 14 d samples, especially at 14 d when there were only six upregulated proteins and five downregulated proteins. Based on the major types of complications during ECMO support as published by the Extracorporeal Life Support Organization (ELSO) (coagulation, hemolysis, infection, inflammation-related, organ damage, etc.) (Supplementary Table S6), the differentially expressed proteins potentially related to ECMO complications were analyzed (Table 5). One upregulated coagulation-related protein (F13A) and five inflammation-related proteins were identified at 7 d on the machine; however, no related protein expressions were observed at 14 d. Proteomic test results showed that, at 7 d on the machine in VV mode, some inflammation-related protein expressions were still abnormal, but they returned to normal levels at 14 d, and no significant biomarkers of protein expression related to ECMO were found.

## 4. Discussion

Given that individuals in need of ECMO devices are often critically ill and in urgent need of treatment, there is considerable uncertainty regarding the recruitment of patients willing to take on the risks involved in clinical trials. To address this, preclinical studies involving sheep are crucial, as their circulatory systems closely resemble that of humans. These studies can effectively replicate the human clinical condition and thus offer vital support for the development and validation of clinical research protocols. In this study, 15 sheep supported by VV-ECMO and VA-ECMO survived to the scheduled end day. All of the sheep were successfully weaned off ECMO without hemolysis, thrombosis, or hemorrhage, and none of the sheep showed symptoms of unconsciousness or dyspnea, suggesting that the LIFEMOTION ECMO device could provide extracorporeal support to the sheep subjects for up to 14 days similar to the control device.

The values of the vital signs are used for evaluating the basic state of patients in intensive care. Previous reported vital signs could be used to access the basic hemodynamics of sheep in ECMO support [1]. In this study, the HR and MAP of sheep supported by LIFEMOTION ECMO were in the normal physiological range, which suggested sheep with LIFEMOTION ECMO had stable hemodynamics during ECMO support. The SO_2_ is normally within the 95–100%, and the SO_2_ of the LIFEMOTION groups was over 97% throughout the trial period. Therefore the LIFEMOTION ECMO device could maintain the sheep’s SO_2_ in the normal range. During VA-ECMO, the sheep of the LIFEMOTION group showed a slightly higher PO_2_ and a slightly lower PCO_2_ and LAC than the control group, suggesting that the sheep of the LIFEMOTION group may have had better oxygenation during VA-ECMO support.

The blood cell count and blood biochemistry could reflect the physiological condition of sheep subjects. The hematology and blood biochemistry values of sheep subjects in the control and LIFEMOTION groups also showed a fluctuation in the normal range, which suggested the LIFEMOTION ECMO device could provide sheep subjects with stable support for maintaining normal physiological function, and the performance of the LIFEMOTION ECMO device in maintaining physiological function was comparable to the control device. In addition, the SaO_2_ is a key conductor of oxygenator performance, and the SaO_2_ was about 100% during ECMO support in the LIFEMOTION groups, comparable to the control device, which suggested the LIFEMOTION ECMO had a satisfactory oxygenation efficiency.

In a clinical setting, patients with ECMO support are at an increased risk of developing significant coagulation and thrombosis, and blood exposed to the artificial surface of the ECMO circuit would result in the activation of the coagulation–fibrinolysis system and inflammatory responses, and coagulation and inflammation are closely linked through humoral and cellular networks, which could lead to intravascular coagulation [26,31]. During ECMO, anticoagulation is necessary to prevent circuit thrombosis or thrombotic complications with heparin [32]. However, the use of heparin might increase the incidence of bleeding events [28]. Therefore, anticoagulation monitoring is essential for achieving optimal anticoagulation during ECMO for patients or trial animals [26,28]. Compared with ACT and aPTT, the anti-Xa concentration directly measures heparin inhibition of factor Xa and is associated with better outcomes in ECMO patients [28,29]. In this study, we monitored coagulation parameters, including activated clotting time (ACT), prothrombin time (PT), activated partial thromboplastin time (APTT), and fibrinogen (FIB). We noticed the ACT value was maintained under 250 s in the normal range, and the values of PT, FIB, and APTT had fluctuations within the pre-operation range during ECMO, which was due to the usage of heparin for anticoagulation. Although no standard targets for anticoagulation exist in clinical practice, the recommended ACT levels for patients are about 180–220 s and under 273 s for sheep [1,33]. Therefore, we reasoned that using heparin during ECMO may influence the coagulation factors and prothrombin time, and the four coagulation indicators within the pre-operation range suggested sheep subjects reserved appropriate anticoagulation management during ECMO.

Proteomics, as a microscopic and precise evaluation method, can evaluate the potential adverse effects of animals on the overall protein expression profile [34,35]. In this study, non-targeted protein quantification techniques were used to perform proteomic sequencing on serum samples of cold-tail sheep on their preoperative day [36], 7 days after surgery, and 14 days after surgery. The results showed that, compared to the preoperative group, there were fewer overall differentially expressed proteins in the groups 7 days and 14 days after surgery, especially in the group 14 days after surgery, where the number of differentially expressed proteins was limited to only a few. Referring to the ECMO complications published by ELSO, the differentially expressed proteins were analyzed to identify potential biomarkers related to ECMO complications. It was found that, in the 7-day on-pump group, regardless of the VA or VV-ECMO, some inflammatory related proteins showed up or were downregulated. However, by the 14th day, the serum protein profile of the animals had basically returned to the preoperative level, and no significant biomarkers related to ECMO were observed. The LIFEMOTION ECMO device demonstrated overall good safety.

The sheep survived until the termination of the trial at 14 days, and we noted there were sheep with histological changes, including necrosis of cardiac muscle, necrosis of renal tissue, and pulmonary infection. In clinical settings, the most common adverse events during ECMO include bleeding, thromboembolism, infection, acute kidney injury, neurologic complications, etc. [26,37,38,39]. Therefore, management during ECMO support is vital to successful weaning in long-term trials of large animals.

Overall, 15 sheep supported with VV-ECMO and VA-ECMO survived for 14 days without transfusion, and these sheep subjects showed stable hemodynamics and physiological function and satisfactory oxygenation performance, as well as proper anticoagulation management during ECMO. The results suggested the LIFEMOTION ECMO device had a comparable safety and efficacy performance to the marketed counterpart.

While sheep are a relatively good animal model for ECMO evaluation, due to species differences, there are still certain differences between sheep and humans in some physiological and biochemical parameters. For example, research has found that there are differences in biochemical and routine blood test indicators between sheep and humans [40]. While ECMO is predominantly employed in clinical settings for patients with critical illnesses, existing international studies predominantly employ healthy animals as experimental subjects. The disease status of animals may also be a crucial determinant for evaluating devices like ECMO in future research. Considering the scarcity of such studies, developing standardized protocols and techniques for this type of research is essential to support future multicenter studies. Therefore, there may be certain limitations in directly extrapolating results from animal experiments to humans. The experimental data from sheep can serve as an important reference for clinical trials.

## 5. Conclusions

In summary, large animal models are highly effective tools for preclinical evaluation of the safety and effectiveness of ECMO. According to the animal trial, the novel ECMO device, LIFEMOTION, demonstrated the safety and acceptable performance of the device by providing satisfactory oxygenation, stable hemodynamics, and physiological function to the sheep. All sheep were successfully weaned from ECMO without transfusion or cannula complications. No significant differences were observed between the two groups in terms of vital signs, oxygenation, hemodynamic stability, and physiological function. No significant biomarkers associated with ECMO clinical complications were identified by proteomic analysis. The LIFEMOTION ECMO device demonstrated overall good safety and efficacy. This study provides preliminary evidence for future clinical trials. This study provides valuable insights for future clinical applications of ECMO devices and investigations into pathological physiology using large animal models.

## Figures and Tables

**Table 1:** Sheep information in ECMO supports.

No	Gender	ECMO Mode	Groups	Duration (Days)	Termination
S1	Male	VV	Control (NOVALUNG XLUNG kit 230)	14	Scheduled
S2	Male	VV	Control (NOVALUNG XLUNG kit 230)	14	Scheduled
S3	Male	VV	LIFEMOTION	14	Scheduled
S4	Male	VV	LIFEMOTION	14	Scheduled
S5	Male	VV	LIFEMOTION	14	Scheduled
S6	Male	VV	LIFEMOTION	14	Scheduled
S7	Male	VV	LIFEMOTION	14	Scheduled
S8	Male	VA	Control (NOVALUNG XLUNG kit 230)	14	Scheduled
S9	Male	VA	Control (NOVALUNG XLUNG kit 230)	14	Scheduled
S10	Male	VA	Control (NOVALUNG XLUNG kit 230)	14	Scheduled
S11	Male	VA	LIFEMOTION	14	Scheduled
S12	Male	VA	LIFEMOTION	14	Scheduled
S13	Male	VA	LIFEMOTION	14	Scheduled
S14	Male	VA	LIFEMOTION	14	Scheduled
S15	Male	VA	LIFEMOTION	14	Scheduled

**Table 2:** Experimental design for grouping.

Sampling Time	VA-ECMO		VV-ECMO	
	Control Group	LIFEMOTION Group	Control Group	LIFEMOTION Group
Pre-operation	2	4	2	5
7 days post-surgery				
14 days post-surgery				

**Table 3:** Alignment strategy between differential groups.

Model	Comparable Group
VA-ECMO	C_VA_D7/C_VA_pre
	C_VA_D14/C_VA_pre
	F_VA_D7/F_VA_pre
	F_VA_D14/F_VA_pre
VV-ECMO	C_VV_D7/C_VV_pre
	C_VV_D14/C_VV_pre
	F_VV_D7/F_VV_pre
	F_VV_D14/F_VV_pre

Note: C_VA_pre refers to the pre-operation sample of the LIFEMOTION group in VA mode, C_VA_D7 refers to the 7-day sample of the LIFEMOTION group in VA mode, C_VA_D14 refers to the 14-day sample of the LIFEMOTION group in VA mode; C_VV_pre refers to the pre-operation sample of the LIFEMOTION group in VV mode, C_VV_D7 refers to the 7-day sample of the LIFEMOTION group in VV mode, C_VV_D14 refers to the 14-day sample of the LIFEMOTION group in VV mode; F_VA_pre refers to the pre-operation sample of the control group in VA mode, F_VA_D7 refers to the 7-day sample of the control group in VA mode, F_VA_D14 refers to the 14-day sample of the control group in VA mode; F_VV_pre refers to the pre-operation sample of the control group in VV mode, F_VV_D7 refers to the 7-day sample of the control group in VV mode, and F_VV_D14 refers to the 14-day sample of the control group in VV mode.

**Table 4:** Proteins potentially associated with VA-ECMO after 14 days support.

Classify	Protein Description	Gene Name	Fold Change	
			7 Days	14 Days
Coagulation	Coagulation factor XIII A chain	F13A1	3.72 (up)	Normal
Hemolysis	Hemoglobin subunit beta	HBB	Normal	4.41 (up)
Infection and Inflammation	Complement C3	C3	4.97 (up)	Normal
	Complement C4-like	C4-like	3.49 (up)	Normal
	Complement factor D	CFD	3.29 (up)	Normal
	Complement C4-A	C4-A	0.09 (down)	Normal
	Interleukin 6 signal transducer	IL6ST	3.49 (up)	Normal
Organic damage	L-lactate dehydrogenase	LDHA	4.01 (up)	Normal
	Creatine kinase	CKM	3.80 (up)	5.65 (up)

**Table 5:** Proteins potentially associated with VV-ECMO after 14 days support.

Classify	Protein Description	Gene Name	Fold Change	
			7 Days	14 Days
Coagulation	Thrombospondin 1	THBS1	3.03 (up)	Normal
Infection and Inflammation	Complement factor D	CD	5.55 (up)	Normal
	Complement C3	C3	4.75 (up)	Normal
	Complement C4-like	C4-like	3.57 (up)	Normal
	Complement C1s	C1s	3.02 (up)	Normal
	Complement C4-A	C4A	0.11 (down)	Normal

## Data Availability

The mass spectrometry proteomics data have been deposited to the iProX with the dataset identifer PXD048630.

## Supplementary Materials

The following supporting information can be downloaded at https://www.mdpi.com/article/10.3390/arm93050034/s1, Supplementary Table S1: The base values of indexes at pre-operation, Supplementary Table S2: The parameter comparation control group with LIFEMOTION group at VV-ECMO model from day 1 to day 7, Supplementary Table S3: The parameter comparation control group with LIFEMOTION group at VV-ECMO model from day 8 to day 14, Supplementary Table S4: The parameter comparation control group with LIFEMOTION group at VA ECMO from day 1 to day 7, Supplementary Table S5: The parameter comparation control group with LIFEMOTION group at VA ECMO from day 8 to day 14, Supplementary Table S6: Possible clinical biomarkers associated with ECMO support [14,41,42,43,44,45,46,47,48,49,50,51,52,53,54,55,56,57,58,59,60,61,62,63,64,65].

## References

[1] Qi J., Gao S., Liu G., Yan S., Zhang M., Yan W., Zhang Q., Teng Y., Wang J., Zhou C. (2021). An Ovine Model of Awake Veno-Arterial Extracorporeal Membrane Oxygenation. Front. Vet. Sci..

[2] Marasco S.F., Lukas G., McDonald M., McMillan J., Ihle B. (2008). Review of ECMO (extra corporeal membrane oxygenation) support in critically ill adult patients. Heart Lung Circ..

[3] Supady A., Combes A., Barbaro R.P., Camporota L., Diaz R., Fan E., Giani M., Hodgson C., Hough C.L., Karagiannidis C. (2022). Respiratory indications for ECMO: Focus on COVID-19. Intensive Care Med..

[4] Milewski R.C., Chatterjee S., Merritt-Genore H., Hayanga J.W.A., Grant M.C., Roy N., Hirose H., Moosdorf R., Whitman G.J., Haft J.W. (2023). ECMO During COVID-19: A Society of Thoracic Surgeons/Extracorporeal Life Support Organization Survey. Ann. Thorac. Surg. Short Rep..

[5] Barbaro R.P., MacLaren G., Boonstra P.S., Iwashyna T.J., Slutsky A.S., Fan E., Bartlett R.H., Tonna J.E., Hyslop R., Fanning J.J. (2020). Extracorporeal membrane oxygenation support in COVID-19: An international cohort study of the Extracorporeal Life Support Organization registry. Lancet.

[6] Schmidt M., Hajage D., Lebreton G., Monsel A., Voiriot G., Levy D., Baron E., Beurton A., Chommeloux J., Meng P. (2020). Extracorporeal membrane oxygenation for severe acute respiratory distress syndrome associated with COVID-19: A retrospective cohort study. Lancet Respir. Med..

[7] Hogen R., Sedra A.H., Motamed A., Emamaullee J. (2021). The evolving role of ECMO in liver transplantation. Curr. Opin. Organ Transpl..

[8] Mou Z., He J., Guan T., Chen L. (2022). Acute kidney injury during extracorporeal membrane oxygenation: VA ECMO versus VV ECMO. J. Intensive Care Med..

[9] Brodie D., Bacchetta M. (2011). Extracorporeal membrane oxygenation for ARDS in adults. N. Engl. J. Med..

[10] Wrisinger W.C., Thompson S.L. (2022). Basics of Extracorporeal Membrane Oxygenation. Surg. Clin. N. Am..

[11] Le Gall A., Follin A., Cholley B., Mantz J., Aissaoui N., Pirracchio R. (2018). Veno-arterial-ECMO in the intensive care unit: From technical aspects to clinical practice. Anaesth. Crit. Care Pain Med..

[12] Nasser M.F., Jabri A., Sharma S., Alhuneafat L., Omar Y.A., Krishnan V., Cameron S.J. (2023). Outcomes with use of extra-corporeal membrane oxygenation in high-risk pulmonary embolism: A national database perspective. J. Thromb. Thrombolysis.

[13] Gerfer S., Djordjevic I., Maier J., Movahed A., Elskamp M., Kuhn E., Liakopoulos O., Wahlers T., Deppe A.C. (2023). Endothelial and Hemodynamic Function in a Large Animal Model in Relation to Different Extracorporeal Membrane Oxygenation Cannulation Strategies and Intra-Aortic Balloon Pumping. J. Clin. Med..

[14] Zhang Y., Peng R., Pei S., Gao S., Sun Y., Cheng G., Yu D., Wang X., Gao Z., Ji B. (2023). Neutrophil extracellular traps are increased after extracorporeal membrane oxygenation support initiation and present in thrombus: A preclinical study using sheep as an animal model. Thromb. Res..

[15] Hartmund Frederiksen P., Linde L., Gregers E., Udesen N., Helgestad O., Banke A., Dahl J., Jensen L., Lassen J., Larsen J. (2023). Cardiac energetics and end-organ perfusion with veno-arterial extracorporeal membrane oxygenation versus ecmella for cardiogenic shock in a large animal model. Eur. Heart J..

[16] Millar J.E., Bartnikowski N., von Bahr V., Malfertheiner M.V., Obonyo N.G., Belliato M., Suen J.Y., Combes A., McAuley D.F., Lorusso R. (2019). Extracorporeal membrane oxygenation (ECMO) and the acute respiratory distress syndrome (ARDS): A systematic review of pre-clinical models. Intensive Care Med. Exp..

[17] Silva K.A.S., Emter C.A. (2020). Large animal models of heart failure: A translational bridge to clinical success. Basic Transl. Sci..

[18] Yates W.G., Schaap R.N., Baumann G.C. (1978). Effects of filler-free silicone rubber on platelets during bovine extracorporeal membrane oxygenation. Trans. Am. Soc. Artif. Intern. Organs.

[19] Djordjevic I., Maier-Trauth J., Gerfer S., Elskamp M., Muehlbauer T., Maul A., Rademann P., Ivanov B., Krasivskyi I., Sabashnikov A. (2023). Fluid Management in Veno-Arterial Extracorporeal Membrane Oxygenation Therapy-Analysis of an Experimental Pig Model. J. Clin. Med..

[20] Karagiannidis C., Joost T., Strassmann S., Weber-Carstens S., Combes A., Windisch W., Brodie D. (2020). Safety and Efficacy of a Novel Pneumatically Driven Extracorporeal Membrane Oxygenation Device. Ann. Thorac. Surg..

[21] Iizuka K., Katagiri N., Takewa Y., Tsukiya T., Mizuno T., Itamochi Y., Kumano K., Tatsumi E. (2018). Evaluation of the Novel Centrifugal Pump, CAPIOX SL, in Chronic Large Animal Experiments. Artif. Organs.

[22] Brekke I.J., Puntervoll L.H., Pedersen P.B., Kellett J., Brabrand M. (2019). The value of vital sign trends in predicting and monitoring clinical deterioration: A systematic review. PLoS ONE.

[23] Jensen L.A., Onyskiw J.E., Prasad N.G. (1998). Meta-analysis of arterial oxygen saturation monitoring by pulse oximetry in adults. Heart Lung.

[24] Wagner P.D. (2015). The physiological basis of pulmonary gas exchange: Implications for clinical interpretation of arterial blood gases. Eur. Respir. J..

[25] Arbiol-Roca A., Imperiali C.E., Dot-Bach D., Valero-Politi J., Dastis-Arias M. (2020). Stability of pH, Blood Gas Partial Pressure, Hemoglobin Oxygen Saturation Fraction, and Lactate Concentration. Ann. Lab. Med..

[26] Millar J.E., Fanning J.P., McDonald C.I., McAuley D.F., Fraser J.F. (2016). The inflammatory response to extracorporeal membrane oxygenation (ECMO): A review of the pathophysiology. Crit. Care.

[27] Gao S., Wang W., Qi J., Liu G., Wang J., Yan S., Teng Y., Zhou C., Wang Q., Yan W. (2021). Safety and Efficacy of a Novel Centrifugal Pump and Driving Devices of the OASSIST ECMO System: A Preclinical Evaluation in the Ovine Model. Front. Med..

[28] Hou X. (2021). Anticoagulation monitoring in extracorporeal membrane oxygenation. Perfusion.

[29] Wilhelmi M.H., Tiede A., Teebken O.E., Bisdas T., Haverich A., Mischke R. (2012). Ovine blood: Establishment of a list of reference values relevant for blood coagulation in sheep. ASAIO J..

[30] Madhani S.P., Frankowski B.J., Burgreen G.W., Antaki J.F., Kormos R., D’Cunha J., Federspiel W.J. (2017). In vitro and in vivo evaluation of a novel integrated wearable artificial lung. J. Heart Lung Transpl..

[31] Zeibi Shirejini S., Carberry J., McQuilten Z.K., Burrell A.J., Gregory S.D., Hagemeyer C.E. (2023). Current and future strategies to monitor and manage coagulation in ECMO patients. Thromb. J..

[32] Levy J.H., Staudinger T., Steiner M.E. (2022). How to manage anticoagulation during extracorporeal membrane oxygenation. Intensive Care Med..

[33] Kato C., Oakes M., Kim M., Desai A., Olson S.R., Raghunathan V., Shatzel J.J. (2021). Anticoagulation strategies in extracorporeal circulatory devices in adult populations. Eur. J. Haematol..

[34] He B., Huang Z., Huang C., Nice E.C. (2022). Clinical applications of plasma proteomics and peptidomics: Towards precision medicine. Proteom.–Clin. Appl..

[35] Li L., Sun C., Sun Y., Dong Z., Wu R., Sun X., Zhang H., Jiang W., Zhou Y., Cen X. (2022). Spatially resolved proteomics via tissue expansion. Nat. Commun..

[36] Gerdle B., Wåhlén K., Gordh T., Bäckryd E., Carlsson A., Ghafouri B. (2022). Plasma proteins from several components of the immune system differentiate chronic widespread pain patients from healthy controls–an exploratory case-control study combining targeted and non-targeted protein identification. Medicine.

[37] Cheng R., Hachamovitch R., Kittleson M., Patel J., Arabia F., Moriguchi J., Esmailian F., Azarbal B. (2014). Complications of extracorporeal membrane oxygenation for treatment of cardiogenic shock and cardiac arrest: A meta-analysis of 1,866 adult patients. Ann. Thorac. Surg..

[38] Xiong J., Zhang L., Bao L. (2020). Complications and mortality of venovenous extracorporeal membrane oxygenation in the treatment of neonatal respiratory failure: A systematic review and meta-analysis. BMC Pulm. Med..

[39] Sun H.-Y., Ko W.-J., Tsai P.-R., Sun C.-C., Chang Y.-Y., Lee C.-W., Chen Y.-C. (2010). Infections occurring during extracorporeal membrane oxygenation use in adult patients. J. Thorac. Cardiovasc. Surg..

[40] Desco M., Cano M.J., Duarte J., Rodriguez F., Fernández-Caleya D., Alvarez-Valdivielso M., Antoranz J.C., Rubio M.A., García-Barreno P., del Cañizo J.F. (1989). Blood biochemistry values of sheep (Ovis aries ligeriensis). Comp. Biochem. Physiol A Comp. Physiol..

[41] Weiss-Tessbach M., Ratzinger F., Obermueller M., Burgmann H., Staudinger T., Robak O., Schmid M., Roessler B., Jilma B., Kussmann M. (2022). Biomarkers for differentiation of coronavirus disease 2019 or extracorporeal membrane oxygenation related inflammation and bacterial/fungal infections in critically ill patients: A prospective observational study. Front. Med..

[42] Schopka S., Philipp A., Müller T., Lubnow M., Lunz D., Unterbuchner C., Rupprecht L., Keyser A., Schmid C. (2020). The impact of interleukin serum levels on the prognosis of patients undergoing venoarterial extracorporeal membrane oxygenation. Artif. Organs.

[43] Liu C.H., Kuo S.W., Ko W.J., Tsai P.R., Wu S.W., Lai C.H., Wang C.-H., Chen Y.-S., Chen P.-L., Liu T.-T. (2017). Early measurement of IL-10 predicts the outcomes of patients with acute respiratory distress syndrome receiving extracorporeal membrane oxygenation. Sci. Rep..

[44] Veraar C., Kirschner E., Schwarz S., Jaksch P., Hoetzenecker K., Tschernko E., Dworschak M., Ankersmit H.J., Moser B. (2022). Follistatin-like 1 and Biomarkers of Neutrophil Activation Are Associated with Poor Short-Term Outcome after Lung Transplantation on VA-ECMO. Biology.

[45] Li J., Yu Z., Zeng J., Liu Z., Zhao Z., Zhang Y., Li G. (2022). Circular RNA UBAP2 (hsa_circ_0007367) Correlates with Microcirculatory Perfusion and Predicts Outcomes of Cardiogenic Shock Patients Undergoing Extracorporeal Membrane Oxygenation Support. Shock.

[46] Coletti K., Griffiths M., Nies M., Brandal S., Everett A.D., Bembea M.M. (2022). Cardiac Dysfunction Biomarkers are Associated with Potential for Successful Separation from Extracorporeal Membrane Oxygenation in Children. Asaio J..

[47] Tsuchida T., Wada T., Gando S. (2021). Coagulopathy Induced by Veno-Arterial Extracorporeal Membrane Oxygenation Is Associated With a Poor Outcome in Patients With Out-of-Hospital Cardiac Arrest. Front. Med..

[48] Siegel P.M., Orlean L., Bojti I., Kaier K., Witsch T., Esser J.S., Trummer G., Moser M., Peter K., Bode C. (2021). Monocyte Dysfunction Detected by the Designed Ankyrin Repeat Protein F7 Predicts Mortality in Patients Receiving Veno-Arterial Extracorporeal Membrane Oxygenation. Front. Cardiovasc. Med..

[49] Ghaleb S., Reagor J.A., Tarango C., Benscoter A., Smith R., Byrnes J.W. (2021). Correlation among Hemolysis Biomarkers in Pediatric Patients Undergoing Extracorporeal Membrane Oxygenation. J. Extra Corpor. Technol..

[50] Pais P., Robinson S., Majithia-Beet G., Lotto A., Kumar T., Westrope C., Sullo N., Eagle H., Bryony B., Joel-David L. (2020). Biomarkers of Inflammation and Lung Recovery in Extracorporeal Membrane Oxygenation Patients With Persistent Pulmonary Hypertension of the Newborn: A Feasibility Study. Pediatr. Crit. Care Med..

[51] Lee A.E., Pandiyan P., Liu M.M., Williams M.A., Everett A.D., Mueller G.P., Morriss M.C., Raman L., Carlson D., Gatson J.W. (2020). Tau Is Elevated in Pediatric Patients on Extracorporeal Membrane Oxygenation. Asaio J..

[52] Yang L., Fan Y., Lin R., He W. (2019). Blood Lactate as a Reliable Marker for Mortality of Pediatric Refractory Cardiogenic Shock Requiring Extracorporeal Membrane Oxygenation. Pediatr. Cardiol..

[53] Zhang Y., Li C.S., Yuan X.L., Ling J.Y., Zhang Q., Liang Y., Liu B., Zhao L.X. (2018). Association of serum biomarkers with outcomes of cardiac arrest patients undergoing ECMO. Am. J. Emerg. Med..

[54] Park S.J., Kim S.P., Kim J.B., Jung S.H., Choo S.J., Chung C.H., Lee J.W. (2014). Blood lactate level during extracorporeal life support as a surrogate marker for survival. J. Thorac. Cardiovasc. Surg..

[55] Schrage B., Rübsamen N., Becher P.M., Roedl K., Söffker G., Schwarzl M., Dreher A., Schewel J., Ghanem A., Grahn H. (2019). Neuron-specific-enolase as a predictor of the neurologic outcome after cardiopulmonary resuscitation in patients on ECMO. Resuscitation.

[56] Wilm J., Philipp A., Müller T., Bredthauer A., Gleich O., Schmid C., Lehle K. (2018). Leukocyte Adhesion as an Indicator of Oxygenator Thrombosis During Extracorporeal Membrane Oxygenation Therapy?. Asaio J..

[57] Tan V.E., Moore W.S., Chopra A., Cies J.J. (2018). Association of procalcitonin values and bacterial infections in pediatric patients receiving extracorporeal membrane oxygenation. Perfusion.

[58] McVey M.J., Kuebler W.M. (2018). Extracellular vesicles: Biomarkers and regulators of vascular function during extracorporeal circulation. Oncotarget.

[59] Chung J.H., Yeo H.J., Kim D., Lee S.M., Han J., Kim M., Cho W.H. (2017). Changes in the levels of beta-thromboglobulin and inflammatory mediators during extracorporeal membrane oxygenation support. Int. J. Artif. Organs.

[60] Lyu L., Long C., Hei F., Ji B., Liu J., Yu K., Chen L., Yao J., Hu Q., Hu J. (2016). Plasma Free Hemoglobin Is a Predictor of Acute Renal Failure During Adult Venous-Arterial Extracorporeal Membrane Oxygenation Support. J. Cardiothorac. Vasc. Anesth..

[61] Omar H.R., Mirsaeidi M., Socias S., Sprenker C., Caldeira C., Camporesi E.M., Mangar D. (2015). Plasma Free Hemoglobin Is an Independent Predictor of Mortality among Patients on Extracorporeal Membrane Oxygenation Support. PLoS One.

[62] Liu C.H., Kuo S.W., Hsu L.M., Huang S.C., Wang C.H., Tsai P.R., Chen Y.S., Jou T.S., Ko W.J. (2016). Peroxiredoxin 1 induces inflammatory cytokine response and predicts outcome of cardiogenic shock patients necessitating extracorporeal membrane oxygenation: An observational cohort study and translational approach. J. Transl. Med..

[63] Bembea M.M., Rizkalla N., Freedy J., Barasch N., Vaidya D., Pronovost P.J., Everett A.D., Mueller G. (2015). Plasma Biomarkers of Brain Injury as Diagnostic Tools and Outcome Predictors After Extracorporeal Membrane Oxygenation. Crit. Care Med..

[64] Astoria M.T., Karam S.E., Moores R.R., Rozycki H.J. (2015). Cardiac Troponin Levels in Neonates Who Require ECMO for Noncardiac Indications Are Elevated in Nonsurvivors. Am. J. Perinatol..

[65] Lubnow M., Philipp A., Dornia C., Schroll S., Bein T., Creutzenberg M., Lehle K. (2014). D-dimers as an early marker for oxygenator exchange in extracorporeal membrane oxygenation. J. Crit. Care.

