# Supplementary material for: The Safety and Performance of a Novel Extracorporeal Membrane Oxygenation Device in a Long-Term Ovine Model
Source: Adv Respir Med. 2025 Sep 9;93(5):34. doi: 10.3390/arm93050034 (PMC12452530; doi:10.3390/arm93050034)
Supplement: Supplementary file 1 [file arm-93-00034-s001.zip › arm-3791972-supplementary.pdf]

Supplementary Material

The Safety and Performance of a Novel Extracorporeal Membrane Oxygenation Device in a Long-term Ovine Model

Yongchao Li[1,2†], Lei Cai[1†], Jia Huang[3†], Hongbin Gao[1], Zhongqiang Huang[1], Yalun Guan[1], Yunfeng Li[1], Shuhua Liu[1], Shi Liang[1], Summer Xiatian Li[4], Hongzhou Lu[3*], Ge Li[1,2*], Yijiang Li[4*], Yu Zhang[1*]

[1] Guangdong Provincial Biotechnology Research Institute (Guangdong Provincial Laboratory Animals
Monitoring Center), No.11, Fengxin Road, Huangpu District, Guangzhou,Guangdong, China, 510663; ycl@gdlami.com (Y.L.); cail@gdlami.com (L.C.); ghb@gdlami.com (H.G.); hzq@gdlami.com (Z.H.); gyl@gdlami.com (Y.G.); lyf@gdlami.com (Y.L.); shuhualiu@gdlami.com (S.L.); liangshi@gdlami.com (S.L.)

[2] Guangzhou National Laboratory, No. 9, Xingdao Ring North Road, Guangzhou International Bio Island, Huangpu District, Guangzhou,Guangdong, China, 510005

[3] National Clinical Research Center for Infectious Diseases, The Third People's Hospital of Shenzhen, 29 Bulan Road, Longgang District, Shenzhen, Guangdong, China, 518112; hjsunnyvale@hotmail.com (J.H.)

[4] Guangdong Organ Support Engineering Technology Research Center, Bldg. 6, Baoxing Wisdom City, No. 650 Zhoushi Road, Bao'An District, 518126 Shenzhen, China, 518126; summerxiatian.li@icloud.com (S.X.L.)

*Correspondence: National Clinical Research Center for Infectious Diseases, The Third People's Hospital of Shenzhen, luhongzhou@fudan.edu.cn (H.L.); Guangzhou National Laboratory, li_ge@gzlab.ac.cn (G.L.); Guangdong Organ Support Engineering Technology Research Center, li.yijiang@hotmail.com (Y.L.); Guangdong Provincial Biotechnology Research Institute (Guangdong Provincial Laboratory Animals
Monitoring Center), zhangyu@gdlami.com (Y.Z.)

† These authors contributed equally to this work.

Supplementary data

Supplementary table S1 The base values of indexes at pre-operation

Index		Mean±Std	(Min,Max)	Median
Vital signs	HR	98.67±18.90	67, 141	100
	MAP	82.47±10.36	67, 102	81
	SaO2	99.53±0.83	97, 100	100
Blood gas	LAC	0.60±0.34	0.2, 1.3	0.5
	PO2	231.60±145.04	100, 509	173
	PCO2	42.68±14.35	15, 60	45
Hematology	WBC	5.06±3.55	0.64, 15.64	4.63
	HGB	104.00±9.09	86, 117	104
	HCT	30.27±3.18	24.8, 36.8	30.2
	PLT	276.60±160.28	38, 603	280
	FHB	0.26±0.17	0.10, 0.65	0.17
Blood biochemistry	TP	58.15±14.23	31.9, 79.5	62.5
	ALB	37.47±10.20	11.5, 56.8	37.6
	ALT	14.27±4.07	5.3, 20.4	13.6
	AST	85.05±22.04	48, 123.8	81.6
	UREA	30.06±13.49	11.53, 49.2	30.22
	LDH	374.51±135.02	24.6, 555.7	395.4
	CRE	108.74±21.81	77.5, 157.5	111.5
	TBIL	1.72±0.66	0.3, 2.9	1.8
	CRP	2.56±4.56	0.61, 18.13	1.03
Coagulation parameters	PT	17.07±5.45	11.9, 31.8	14.6
	APTT	63.24±59.88	1.41, 205.2	38.5
	FIB	0.85±0.28	0.528, 1.674	0.786
	ACT	221.93±69.82	123, 350	204

Supplementary table S2 The parameter comparation control group with LIFEMOTION group at VV-ECMO model from day 1 to day 7.

Index			Day 1	Day 2	Day 3	Day 4	Day 5	Day 6	Day 7
Hematology	WBC	Control	6.76±4.19	11.03±5.21	9.77±0.62	8.23±2.84	8.11±2.05	8.38±0.46	7.30±1.41
		LIFEMOTION	7.05±1.40	12.32±2.81	11.98±2.54	11.92±3.33	11.88±2.57	12.98±4.04	14.64±4.83*
	HGB	Control	106.12±14.02	93.45±12.80	89.75±2.47	87.75±1.06	89.25±4.60	90.75±7.42	87.25±3.89
		LIFEMOTION	109.56±10.84	97.25±4.19	90.90±12.83	86.90±16.42	89.30±18.33	84.60±18.42	82.90±19.88
	HCT	Control	30.92±3.68	27.18±3.42	26.08±0.74	25.95±0.00	26.50±1.56	26.92±2.51	25.88±1.80
		LIFEMOTION	32.39±4.67	29.23±2.24	27.02±3.24	25.93±4.80	26.68±5.21	26.64±3.42	24.22±5.91
	PLT	Control	322.79±159.04	281.90±146.65	223.25±84.50	301.25±181.37	394.50±132.23	466.50±39.60	483.00±33.94
		LIFEMOTION	188.46±137.61	173.88±114.69	175.70±126.40	287.60±80.29	348.10±160.23	517.60±237.89	532.20±211.65
	FHB	Control	0.25±0.00	0.20±0.02	0.24±0.04	0.25±0.12	0.28±0.12	0.29±0.04	0.26±0.00
		LIFEMOTION	0.23±0.03	0.23±0.06	0.28±0.03	0.25±0.02	0.26±0.05	0.31±0.17	0.25±0.02
Blood biochemisty	ALB	Control	42.18±2.73	37.54±1.43	36.65±2.12	39.50±1.34	39.55±2.76	41.03±3.78	40.72±2.72
		LIFEMOTION	32.46±0.11	32.35±2.85*	36.89±6.91	35.17±4.71	35.64±4.12	32.85±4.83	34.01±5.82
	ALT	Control	21.89±8.97	36.02±25.82	51.12±41.47	48.83±38.86	42.20±31.54	36.35±22.27	29.80±16.76
		LIFEMOTION	24.52±12.22	40.65±35.43	47.26±38.61	45.17±21.58	36.91±19.81	31.36±14.80	30.25±11.81
	TP	Control	64.70±8.85	54.81±2.10	58.77±7.25	53.83±6.05	48.62±14.74	49.62±15.87	50.80±15.56
		LIFEMOTION	44.42±15.28	48.12±11.72	64.10±10.59	60.73±3.72	62.78±5.83	54.59±9.56	51.73±12.13
	AST	Control	127.79±13.53	238.52±126.55	314.27±222.49	285.70±174.66	229.80±116.60	180.35±79.90	141.98±42.96
		LIFEMOTION	219.34±148.18	374.20±339.64	431.28±357.48	384.72±131.87	324.73±164.25	260.37±100.06	237.09±94.16
	UREA	Control	27.83±12.37	23.95±4.40	24.00±5.32	34.12±1.94	34.45±3.51	37.79±0.46	39.37±4.17
		LIFEMOTION	33.05±13.53	26.63±14.02	21.70±7.61	20.95±7.16*	25.11±8.16	33.09±12.19	36.55±12.36
	LDH	Control	606.64±122.58	974.40±346.77	912.70±311.27	823.55±297.62	669.40±158.25	599.58±49.96	528.35±20.44

Index		Day 1	Day 2	Day 3	Day 4	Day 5	Day 6	Day 7
	LIFEMOTION	619.69±257.55	856.31±637.18	861.60±376.61	849.92±146.69	790.30±210.32	761.19±268.78	685.31±236.79
SBb	Control	1.55±0.49	1.65±0.49	2.40±0.71	2.10±0.28	1.05±0.21	1.45±0.21	1.50±0.14
	LIFEMOTION	2.18±0.56	2.36±0.73	3.10±0.98	2.96±0.97	2.38±1.04*	2.26±1.29	2.04±1.26
CRE	Control	121.72±5.39	118.56±29.50	114.35±42.92	164.57±84.46	93.33±39.70	116.43±5.83	106.97±15.52
	LIFEMOTION	102.26±5.97*	95.20±4.56	114.47±5.43	90.25±18.44	77.42±23.55	82.84±16.94*	90.39±3.95

Supplementary table S3 The parameter comparation control group with LIFEMOTION group at VV-ECMO model from day 8 to day 14.

Indexes		Groups	Day 8	Day 9	Day 10	Day 11	Day 12	Day 13	Day 14
Hematology	WBC	Control	7.58±0.98	11.32±1.60	11.79±3.38	10.30±0.13	9.31±1.57	9.66±0.08	7.89±0.72
		LIFEMOTION	15.92±5.79*	17.18±5.69	19.99±5.57	18.81±5.80*	17.62±7.54	18.85±11.17	15.68±10.43
	HGB	Control	89.50±6.36	95.75±6.01	99.75±4.60	90.00±0.00	90.00±2.12	94.25±8.13	89.00±8.49
		LIFEMOTION	81.90±25.28	83.10±24.37	90.60±15.59	86.50±20.12	92.40±18.20	90.10±15.84	97.10±12.51
	HCT	Control	26.43±2.16	27.95±2.47	29.00±0.35	26.23±0.81	26.25±0.99	27.02±2.58	26.02±1.80
		LIFEMOTION	23.64±7.58	24.75±6.48	27.23±4.48	26.03±6.29	26.23±6.33	25.63±5.90	28.67±3.10
	PLT	Control	577.75±8.13	552.50±20.51	443.00±5.66	424.00±2.83	369.50±20.51	313.75±6.01	325.00±72.12
		LIFEMOTION	488.60±180.57	487.40±237.60	441.40±134.09	412.70±206.84	300.50±139.52	267.10±148.00	302.70±253.50
Blood biochemistry	FHB	Control	0.30±0.04	0.25±0.03	0.32±0.04	0.31±0.04	0.29±0.18	0.36±0.01	0.35±0.05
		LIFEMOTION	0.24±0.03	0.30±0.05	0.34±0.04	0.35±0.06	0.31±0.11	0.35±0.13	0.35±0.07
	TP	Control	66.55±4.38	69.08±3.64	68.40±0.49	67.00±0.78	69.97±2.72	67.78±5.90	65.58±7.25
		LIFEMOTION	53.33±12.02	56.83±12.45	62.83±8.55	61.23±13.52	52.93±14.17	65.89±7.18	63.42±15.18
	ALB	Control	41.08±0.18	44.60±2.62	44.90±2.05	42.85±0.92	42.28±1.31	43.58±1.73	43.60±1.13
		LIFEMOTION	32.99±5.38*	35.53±5.35*	36.16±5.60*	36.09±5.30*	34.20±9.36	36.52±5.14*	37.36±10.24
	ALT	Control	22.05±14.71	20.73±11.14	18.05±10.82	15.60±5.66	14.68±2.93	18.77±4.63	13.70±1.56
		LIFEMOTION	27.07±9.67	24.62±7.33	24.15±9.47	23.93±8.61	21.34±9.05	23.56±7.91	24.50±9.40
	AST	Control	114.60±50.06	111.25±18.81	100.30±11.53	82.33±2.37	84.70±12.87	100.73±46.92	85.42±30.51
		LIFEMOTION	192.25±73.47	179.91±49.72*	210.73±102.33	187.71±93.65	150.24±58.28	159.61±62.27	203.81±149.58
	UREA	Control	48.06±2.75	49.11±8.74	55.55±0.52	53.03±5.19	53.80±6.05	51.12±8.81	52.12±5.72
		LIFEMOTION	37.83±8.78	37.76±12.37	38.19±18.04	43.65±18.74	42.42±16.45	43.33±18.50	45.27±21.19
	LDH	Control	529.88±144.57	587.75±2.40	606.17±27.12	497.62±42.11	513.62±65.16	583.15±198.63	515.83±154.54

Indexes	Groups	Day 8	Day 9	Day 10	Day 11	Day 12	Day 13	Day 14
	LIFEMOTION	681.55±143.61	672.68±123.45	811.30±274.59	730.58±204.35	556.97±115.37	653.96±205.45	652.67±194.45
SBb	Control	100.58±19.91	115.88±13.26	115.25±18.24	114.83±25.42	106.75±22.63	108.78±20.61	109.62±21.53
	LIFEMOTION	83.26±3.24	79.92±6.62	68.39±28.40	90.64±7.91	74.33±25.39	92.64±10.38	94.19±13.59
CRE	Control	13.10±0.57	13.90±0.21	13.77±1.59	13.82±1.52	14.80±1.70	14.48±3.36	14.10±2.83
	LIFEMOTION	17.17±5.71	20.00±10.68	22.52±13.51	19.19±8.48	20.59±6.36	24.25±7.77	21.91±13.84

Supplementary table S4 The parameter comparation control group with LIFEMOTION group at VA ECMO from day 1 to day 7.

Index			Day 1	Day 2	Day 3	Day 4	Day 5	Day 6	Day 7
Hematology	WBC	Control	7.10±2.28	11.60±0.44	14.44±9.38	11.52±7.59	9.30±3.16	10.37±4.94	10.92±5.89
		LIFEMOTION	6.08±1.11	10.71±3.35	9.14±2.55	9.31±1.69	10.40±2.55	11.89±3.23	12.25±3.47
	HGB	Control	105.57±4.13	109.88±7.64	99.17±5.86	93.50±9.54	95.67±11.12	102.00±17.76	99.33±10.56
		LIFEMOTION	102.42±11.69	94.88±11.21	87.50±10.48	87.00±5.33	85.40±4.34	84.10±3.47	85.60±6.23
	HCT	Control	31.68±3.75	30.40±2.87	28.45±2.01	27.28±4.05	28.17±4.29	28.10±3.73	27.72±2.79
		LIFEMOTION	29.53±3.54	27.62±3.36	25.31±2.74	25.55±1.29	24.86±0.94	24.33±1.24	25.15±1.94
	PLT	Control	291.47±45.57	232.04±25.07	242.50±62.19	232.00±45.07	267.50±51.57	348.17±84.04	375.17±88.26
		LIFEMOTION	121.54±53.96*	103.34±51.08*	99.50±54.23*	145.50±101.22	232.70±196.01	257.60±215.93	247.20±167.14
	FHB	Control	0.25±0.02	0.20±0.03	0.32±0.07	0.28±0.05	0.23±0.11	0.30±0.08	0.25±0.01
		LIFEMOTION	0.25±0.03	0.26±0.06	0.23±0.05	0.24±0.04	0.28±0.05	0.25±0.09	0.22±0.10
Blood biochemistry	TP	Control	59.02±7.00	55.43±6.62	59.07±8.35	55.47±12.81	57.18±10.95	55.63±18.14	56.95±16.61
		LIFEMOTION	54.83±8.12	52.21±6.49	60.14±12.51	69.30±10.08	64.26±5.64	62.87±6.52	65.15±25.02
	ALB	Control	39.34±5.87	36.58±7.80	38.20±6.61	37.02±4.77	37.27±4.57	39.72±5.20	40.32±6.86
		LIFEMOTION	34.20±7.08	32.40±2.92	35.75±7.26	44.22±13.34	36.51±4.15	36.68±5.21	35.10±6.72
	ALT	Control	25.34±6.82	37.02±10.93	39.58±5.11	35.05±5.91	25.02±7.06	26.80±5.48	25.53±9.20
		LIFEMOTION	21.12±7.10	33.21±13.96	45.87±24.36	45.95±23.37	41.98±18.90	38.06±22.47	29.12±17.02
	AST	Control	199.76±53.53	309.30±90.57	315.37±48.92	272.00±53.94	181.43±26.16	211.52±50.15	204.23±64.51

Index		Day 1	Day 2	Day 3	Day 4	Day 5	Day 6	Day 7
	LIFEMOTION	171.65±59.89	273.05±114.07	361.06±182.86	318.10±126.59	306.53±153.76	295.72±176.80	225.03±144.52
UREA	Control	32.91±6.23	32.68±4.43	28.62±4.47	34.65±1.95	39.33±3.70	42.94±8.89	47.56±4.13
	LIFEMOTION	31.75±16.24	31.70±15.10	28.93±15.01	36.22±19.59	35.70±21.13	36.25±20.53	39.92±22.51
LDH	Control	713.28±270.30	1011.4±435.3	678.90±190.88	694.95±176.46	570.02±172.10	487.53±57.01	669.47±189.54
	LIFEMOTION	660.91±117.57	973.75±268.53	938.90±314.81	916.97±137.37	868.14±170.19	911.32±349.86	659.95±328.20
SBb	Control	1.97±0.45	1.80±1.47	4.73±5.05	2.07±2.15	2.87±1.11	2.77±2.06	3.37±3.15
	LIFEMOTION	1.96±0.53	2.26±0.39	3.14±0.76	2.36±0.93	1.80±0.69	1.66±0.83	2.10±0.27
CRE	Control	123.54±40.47	126.78±49.13	89.08±20.65	95.20±14.95	82.17±36.68	100.07±21.27	101.18±21.08
	LIFEMOTION	118.33±20.45	121.41±12.63	118.54±17.61	118.91±24.75	120.44±31.98	126.99±45.50	101.13±41.82

Supplementary table S5 The parameter comparation control group with LIFEMOTION group at VA ECMO from day 8 to day 14.

Index			Day 8	Day 9	Day 10	Day 11	Day 12	Day 13	Day 14
Hematology	WBC	Control	13.53±7.41	16.36±8.01	17.04±6.98	19.46±11.07	19.88±11.08	17.26±14.36	18.38±14.52
		LIFEMOTION	10.60±1.43	11.57±2.28	10.08±2.62	10.80±2.86	10.75±3.13	11.26±4.65	10.14±3.53
	HGB	Control	95.67±8.33	93.67±4.04	97.00±8.41	99.33±11.86	94.67±2.31	96.33±5.53	98.17±2.75
		LIFEMOTION	81.60±7.07	90.20±10.19	83.90±6.81	84.00±10.78	83.70±12.47	82.77±13.71	85.25±12.65
	HCT	Control	26.85±1.73	27.65±3.01	28.48±3.54	26.47±1.32	27.33±1.15	27.37±2.16	28.28±1.92
		LIFEMOTION	24.71±2.63	26.56±3.11	24.42±2.23	25.17±3.39	24.61±4.22	24.73±4.32	25.89±4.32
	PLT	Control	366.17±109.99	351.50±102.90	319.83±95.42	312.83±157.54	313.33±200.17	317.50±223.60	317.33±231.62
		LIFEMOTION	213.80±134.35	156.90±58.63	202.50±129.12	169.10±89.20	161.70±102.73	196.03±137.77	201.83±124.37
	FHB	Control	0.28±0.05	0.32±0.08	0.28±0.06	0.34±0.07	0.33±0.01	0.40±0.02	0.38±0.11
		LIFEMOTION	0.25±0.06	0.26±0.07	0.27±0.02	0.31±0.15	0.35±0.15	0.31±0.11	0.37±0.12
Blood biochemistry	TP	Control	58.25±7.11	64.18±1.40	66.38±2.01	61.32±9.59	64.50±4.01	61.78±7.20	63.67±9.36
		LIFEMOTION	56.32±16.23	63.04±20.09	57.80±21.43	58.75±23.96	48.89±22.48	61.66±23.51	60.44±23.23
	ALB	Control	39.32±6.47	39.92±6.30	40.63±6.31	34.83±15.70	41.17±5.76	41.05±6.04	42.67±8.06
		LIFEMOTION	42.88±18.59	38.30±5.29	32.92±11.03	36.93±7.82	37.41±4.16	37.40±11.86	38.73±7.55
	ALT	Control	30.45±14.80	28.85±13.80	35.62±13.74	30.13±13.28	32.72±20.13	35.22±28.29	33.00±26.07
		LIFEMOTION	26.22±5.15	35.82±30.31	24.33±13.51	21.92±13.24	20.90±9.06	21.58±10.03	26.75±8.41
	AST	Control	232.57±94.74	237.28±107.48	271.68±137.52	230.07±123.27	258.88±147.28	275.12±172.34	256.05±155.40
		LIFEMOTION	209.33±69.18	220.94±106.58	174.44±123.69	180.34±105.24	188.26±102.25	223.51±125.29	163.80±144.79
	UREA	Control	52.54±0.51	52.00±1.36	55.69±2.09	51.15±6.39	54.65±2.69	55.03±4.74	56.06±5.88
		LIFEMOTION	39.75±22.63	43.28±18.40	44.12±20.72	43.87±20.32	46.78±21.30	39.93±23.68	**27.58±15.27***
	LDH	Control	822.27±246.86	873.12±349.43	970.43±339.54	780.70±261.92	929.20±547.49	1113.83±784.50	1008.23±659.15
		LIFEMOTION	683.77±261.90	673.27±165.03	687.52±387.19	697.08±365.10	705.69±139.76	654.58±196.55	572.09±301.46

Index			Day 8	Day 9	Day 10	Day 11	Day 12	Day 13	Day 14
	SBb	Control	3.33±2.49	2.73±1.67	2.90±3.42	2.63±4.13	2.77±3.18	1.67±0.81	1.80±0.85
		LIFEMOTION	1.94±0.90	2.12±1.22	2.78±0.89	2.84±0.66	2.76±1.15	2.64±0.80	1.24±0.59
	CRE	Control	95.15±19.43	92.65±18.80	83.40±19.86	88.20±20.28	95.75±14.28	98.82±10.32	92.18±15.21
		LIFEMOTION	111.81±54.50	122.80±50.66	106.00±67.22	106.04±38.56	127.03±57.20	119.57±61.39	81.12±16.36

Supplementary table S6 Possible clinical biomarkers associated with ECMO support.

**Markers**	**Functional description**	**Reference**
NETs（Neutrophil extracellular traps）	Neutrophil extracellular traps are increased after extracorporeal membrane oxygenation support initiation and present in thrombus.	**[41]**
IL-6, IL-8 and IL-10	Associated inflammation	**[42-44]**
LCN2, CCL-4 and FSTL1	Significantly correlated with poor prognosis in lung transplantation.	**[45]**
RNA UBAP2(hsa_circ_0007367)	There is a significant correlation between relative expression levels and 30-day in-hospital mortality rate.	**[46]**
ST2 (tumorigenicity 2)	ST2 can be used as a biomarker for extubation and weaning off extracorporeal membrane oxygenation (ECMO) in children.	**[47]**
prothrombin time ratio and antithrombin	Strong correlation to poor prognosis in VAECMO patients.	**[48]**
Mac-1（leukocyte integrin Mac-1 ）	Predicting potential risk of death in ECMO patients.	**[49]**
AST and LDH	Plasma AST and LDH can be used as quantitative indicators for hemolysis.	**[50]**

Platelet-leucocyte aggregation	Patients who died or required prolonged extracorporeal membrane oxygenation support demonstrated elevated levels of platelet-leucocyte aggregation.	**[51]**
Tau	Significant elevation of Tau protein levels in ECMO patients.	**[52]**
Serum lactate	Serum lactate is a reliable marker for mortality in pediatric refractory cardiogenic shock treated with ECMO.	**[53] [54, 55]**
NSE (neuron-specific enolase)	Neuron-specific aldehyde oxidase can be used to assess neurological prognosis following ECMO.	**[56]**
Leukocyte adhesion	The aggregation of leukocytes on the gas exchange membrane of the membrane oxygenator may lead to thrombus formation and impact prognosis.	**[57]**
PCT (procalcitonin)	A PCT value of 0.5 ng/mL can serve as a highly sensitive biomarker for determining whether ECMO patients have a bacterial infection.	**[58]**
Evs (extracellular vesicles)	Extracellular vesicles (EVs) can serve as biomarkers for evaluating vascular function post-ECMO treatment.	**[59]**
βTG (β-thromboglobulin)	Related to platelet dysfunction.	**[60]**
PFHb (plasma free hemoglobin)	Free hemoglobin in adult plasma is a biomarker of acute kidney injury during VA-ECMO.	**[61, 62]**
Prdx1 (Peroxiredoxin)	Inflammatory cytokine response caused by Peroxiredoxin 1 is a biomarker for the prognosis of cardiogenic shock ECMO.	**[63]**
Brain injure	High levels of glial fibrillary acidic protein (GFAP) during ECMO support are significantly associated with acute brain injury and mortality.	**[64]**
cTnl	cTnl is an independent biomarker of poor prognosis in neonatal ECMO.	**[65]**
D-dimer	D-dimers as an early marker for oxygenator exchange in extracorporeal membrane oxygenation.	**[66]**
